# Cortical Substrate of Supraspinal Fatigue following Exhaustive Aerobic Exercise Localizes to a Large Cluster in the Anterior Premotor Cortex

**DOI:** 10.3389/fneur.2017.00483

**Published:** 2017-09-21

**Authors:** Priyantha Herath, Martin Carmichael, Angela Murphy, Leonardo Bonilha, Roger Newman-Norlund, Chris Rorden, Mark Davis

**Affiliations:** ^1^Department of Neurology, School of Medicine, University of South Carolina, Columbia, SC, United States; ^2^Department of Physical Education and Exercise Studies, Lander University, Greenwood, SC, United States; ^3^Department of Pathology, Microbiology and Immunology, School of Medicine, University of South Carolina, Columbia, SC, United States; ^4^Department of Neurology, Medical University of South Carolina, Charleston, SC, United States; ^5^Division of Applied Physiology, Department of Exercise Science, Arnold School of Public Health, University of South Carolina, Columbia, SC, United States; ^6^Department of Psychology, University of South Carolina, Columbia, SC, United States

**Keywords:** central fatigue, muscle contraction, neural representation, functional magnetic resonance imaging, blood oxygen-dependent, anterior premotor cortex

## Abstract

Strenuous exercise leads to a progressive reduction in the performance of voluntary physical exercise. This is due to a process described as fatigue and is defined as the failure to maintain the required or expected power output. While some of this is muscular in origin, there are data suggestive of how fatigue is modulated by cortical signals, leading to a concept of central fatigue. The previously reported fatigue-induced changes in cortical activity may have been due to blood oxygen-dependent (BOLD) signal drift and/or neural habituation alone. We implemented a functional magnetic resonance imaging paradigm to effectively isolate brain areas responsible for central (supraspinal) fatigue following exercise. Our data identify a large cluster that includes dominant the anterior ventral premotor cortex (aPMv), the insula and postcentral gyrus as critical nodes in the brain network where supraspinal fatigue might have their functional neural imprints. Findings here show that activity in the ipsilateral aPMv and the adjacent areas in the premotor cortex correlates with both localized fatigue (fatigue specific hand grip contraction), and generalized full body exhaustive fatigue. In addition, from a methodological standpoint, we have also shown that the effects of BOLD signal drift can be modeled and removed to arrive at specific brain activity patterns in our experiments. Once the loci of central fatigue are isolated in this way, treatments aimed at modulating activity in these premotor areas may reduce exercise-induced fatigue and perhaps also benefit various clinical conditions in which fatigue is a major symptom.

## Introduction

Fatigue, defined as the failure to maintain the required or expected power output, is a ubiquitous human experience. It is reasonable to presume that it is perhaps a fundamental phenomenon in animal physiology. Even amongst the healthy, fatigue limits physical performance during activities of daily life. More importantly, fatigue is a major problem in many disease conditions that range from psychiatric, to cancer and also includes various neurological conditions such as multiple sclerosis and parkinsonism. In all of them, fatigue is extremely common, overpowering symptom that often tends to have a profound negative impact on patients’ motivation, adherence to treatment regimens, their sense of wellbeing, and ultimately, their quality of life. Community and primary care studies estimate 5–45% of the population report fatigue as a debilitating symptom and 2–11% report fatigue lasting at least 6 months (1). Based on decades of physiological investigations, there appears to be two components to fatigue. Of these, peripheral fatigue is fairly well understood and is thought to be due to perturbations that occur in the surface membrane of the muscle, excitation-contraction coupling within individual muscle fibers, and subsequent metabolic events that occur during repetitive contraction-relaxation cycle that result in muscle activity leading to mobility of the animal (2). The other component is called the central or supraspinal or the central fatigue (3, 4). This type of fatigue is thought to occur when there is a measurable decrease in muscle force attributable to a decline in motor-neuronal output. Formal investigation of central or supraspinal fatigue has received limited attention and is the focus of the present work. An additional dichotomy can be drawn between localized fatigue (e.g., cutting a rope with scissors for the 1,000th time in an hour) vs. generalized full body fatigue (e.g., cutting a rope with scissors after an arduous bicycle ride). The paradigm we report here attempts to model both dichotomies.

How the brain might perceive and modulate fatigue is currently unknown. Given that not much is known about the cortical representation of fatigue most ideas about fatigue tend to be conjectural in nature. However, a few fundamental ideas are well characterized. In general, with repetitive excitation and coupling, the ability to generate force in muscles decline (5) which is often what is described as “fatigue.” This initial decline of force during exercise is due to mechanisms within the contracting muscles (5) and then prompt fed back to the brain, and the brain must somehow adjust its activity to maintain the required force output by the muscles. How this adjustment is made at the cortical level or how the sensory information affects the cortical output, is unknown. In general, several domains are likely contributory to the sensation of fatigue, and perhaps they all work in concert to induce what might be eventually detected as fatigue by animals including humans. These could include muscle homeostatic factors, peripheral factors and central factors, and psychological factors, each with their own, and perhaps overlapping neural substrates.

So far, several investigations have reported a reduction in the neural signals responsible for driving working muscles in fatigued subjects (6–8). In the muscles themselves, repetitively sustaining maximal or near-maximal force, the force drops to 50% of its initial value in about 1 min, and almost all the loss in force is due to mechanisms within the contracting muscles (3). When transcranial magnetic stimulation (TMS) is used to modulate the activity of the primary motor cortex (M1) during fatigue, maximal voluntary contractions (MVCs) enhanced by TMS to M1 declines less significantly than unenhanced MVCs (4). This finding conclusively demonstrates the role of central or supra-spinal fatigue in motor output. Additionally, neural output from M1 is thought to be reduced during fatigue (8) with an estimated 30% of muscle fatigue originating from the cortical region. Given that all peripheral phenomena are ultimately under central control, it is then conceivable that the brain has a large contribution toward how we perceive fatigue. Identification of the cortical mapping of central representation of fatigue is important, not only for further understanding what fatigue might actually be, but also in answering whether fatigue serves a beneficial role which can be potentially developed for therapeutic interventions aimed at alleviating excessive fatigue in patients.

There is now a consensus that introspectively perceived (and perhaps many non-sensate) fatigue phenomena are represented cortically as network activity (9, 10). These can be measured indirectly by way of their metabolic surrogates, for example, with functional brain imaging techniques such as functional magnetic resonance imaging (fMRI) and positron emission tomography or single-photon emission computed tomography. When used to study fatigue, two main findings have emerged from the existing fMRI fatigue literature; cerebral metabolism appears to increase during fatiguing exercise (dFEX) (4, 11). This is followed by a decrease in brain metabolism after the fatiguing exercise (aFEx) (12, 13). In another study the fMRI signal increased significantly during the first minute of the contraction and then decreased (5) suggesting the possibility that the loss of force generated is due to increased inhibition from group III and IV afferents that convey information from pain and other sensory receptors. These provocative findings have raised the question whether cortical representation of fatigue is global, rather than merely representing local (affecting only those areas involved in motor control) alterations in the metabolic demand.

It is well known that the signal measured by fMRI is relative and plagued by low-frequency drift. Sustained scanning leads to a progressive warming of the scanner’s components (gradient heating), which can result in gradual changes in signal intensity (14). Furthermore, participant head motion influences field homogeneity, potentially leading to apparent signal changes (15), and head motion is known to increase during fatigue (16). We postulate that previous reports of cortical blood oxygen-dependent (BOLD) signal changes due to fatigue are unable to distinguish between these artifacts and brain metabolism. Additionally, it is possible that previous reports may also reflect the normal neural habituation that is seen when an individual repeats any motor task, even in tasks that lack muscular fatigue (17).

Our aim was to replicate previous findings while controlling for these potential confounds. To address these questions, we have used a novel fMRI paradigm that allowed us to control for drift and effects of habituation while imaging fatigue. Specifically, we measured the amplitude of neural responses evoked by weak motor movements relative to rest. By looking at these transient, high frequencies responses, we address the issue of low frequency drift. During each day of scanning, we measured contractions of the left hand (LH) and right hand (RH) both before, during and after one of the hands was fatigued. This provides a measure for local fatigue (LF). Next, each individual was scanned on different days, following either fatiguing or light exercise. This allows us to examine whether generalized fatigue leads to different amplitude responses.

## Methods

### Participants

Data were collected from 12 volunteers, all students at the University of South Carolina (4 females, 8 males, mean age 24 years, SD = 6). All of these participants took part in experiment 1, and five of them continued to take part in experiment 2 (one female, four males, mean age 28 years, SD = 6). All participants were RH dominant (Handedness inventory) and possessed normal vision. None of the participants reported any history of mental abnormality. For each experiment, participants volunteered for two sessions, each of which required approximately 2 h of commitment. All procedures were approved by the institutional review board of the University of South Carolina and were in accordance with the Declaration of Helsinki.

### Determination of Handgrip MVC

Maximal handgrip strength for both hands was determined using a Smedley-type handgrip dynamometer (Therapeutic Instruments, Clifton, NJ, USA). After subjects were instructed on the proper use of the device they were then instructed to perform three maximal force contractions after receiving a signal while seated with arms by their sides. Subjects paused for 30 s between the three allowed attempts. The best of these three contractions was considered their MVC force.

### Determination of VO2max

To determine maximal aerobic capacity (VO2max), all participants performed an incremental cycle ergometer protocol on a Monarch 818E bike ergometer with expired gas analysis. Testing was begun at 60W/70W which was increased by 30W/75W every 3 min stage for females and males, respectively. If a subject performed past a fifth stage, wattage was increased every minute. Gas analysis was performed on a Moxus Modular VO2 System (AEI Technologies, Inc., Naperville, IL, USA). Prior to beginning the test, subjects were instructed on the use of a rating scale of perceived exertion (RPE). Heart rate was recorded the last 15 sec of each stage while RPE was recorded at the beginning of the final minute of each 3 min stage. VO2max were determined whether: (1) VO2 values did not increase with a subsequent increase in workload; (2) HR > 90% age-predicted maximal heart rate; (3) RER > 1.1; and (4) a RPE > 17.

### Ride to Fatigue

Three days after their VO2max test, subjects underwent a 90-min ride to fatigue on a bicycle ergometer at 60% maximal power output that was attained during the final full stage of their VO2max test. During this ride, RPE were recorded along with heart rate. Fatigue was called when subjects reported RPE values >17 and were no longer able to maintain the proper pedaling cadence for a period of 30 s.

## Experimental Procedures

### Generalized Full-Body Fatiguing Protocol

One week following the preliminary ride to fatigue session and prior to each day’s scanning, the participants completed 90 min of exercise on a bicycle ergometer in the laboratory adjacent to the scanner. Cycling was performed on a Monarch 818E bike ergometer modified to include an SRM Power meter (SRM International, SRM GmbH, Deutschland). Subjects wore a Polar heart rate transmitter. The transmitter’s signal was detected by the SRM Power Control (SRM International, SRM GmbH, Deutschland). Heart rate was recorded every 15 min. On one of the days, cycling was performed at a low (30% VO2max; easy—Ẹ easy ride). On another day, the cycling intensity was individually calibrated to achieve complete physical exhaustion, or generalized full body fatigue (Ḥ hard ride). To accomplish this, subjects began the cycling bout at 60% of the max power output that was attained during the final full stage of their VO2max test. They then maintained that effort for 70 min. Over the last 20 min, cycling intensity was individually adjusted by increasing the wheel resistance slightly to ensure that they reached a state of fatigue. Fatigue was determined in the same manner as during the preliminary ride to fatigue. Importantly, the total number of pedal revolutions during the 90 min ride was strictly controlled so that the only difference between sessions was that the ride on 1 day (h) produced fatigue, while the ride on the other day (e) did not (i.e., intensity was manipulated by altering the pedaling resistance). Subjects rested for 10 min between cycling and brain imaging, during which blood pressure and heart rate were monitored to ensure that participants returned to their precycling baseline conditions before scanning.

### Scanning Protocol

**Figure 1:**
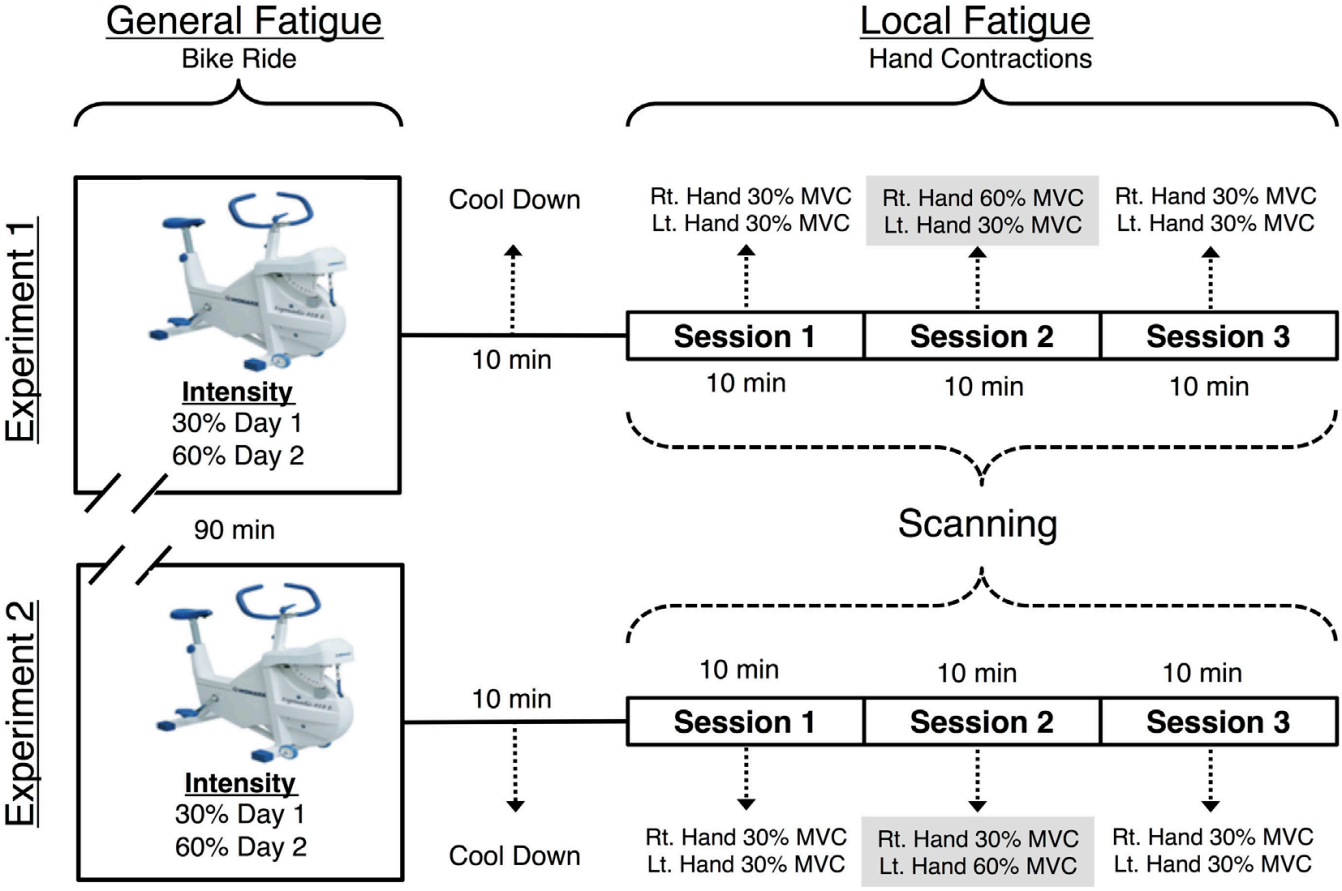
Visual representation of the functional magnetic resonance imaging (fMRI) paradigm. Each individual was scanned on 2 days, with a 90 m cycle ride preceding the MRI scan (with hard exertion on one day and easy exertion on the other). The MRI scan included three 616 s long sessions. During the first, before fatigue session the participant made hand contractions that were of moderate and equal difficulty for the left and right hands. During the second, fatiguing session, one hand was required to make exhausting contractions. The final, after fatigue session returned to moderate contractions for both hands. During each session the participant made 72 contractions of the hand to be fatigued, 72 contractions of the non-fatigued hand and observed 72 visual trials that required no response.

We conducted two separate experiments (see Figure 1 for the schematic). In each of these experiments, participants were scanned on 2 days. On day 1, participants were scanned after a non-fatiguing, easy ride (Ë) on a bicycle ergometer, while on day 2, they underwent scanning after an exhaustive hard ride (Ḥ) on the bicycle ergometer. Generalized full body fatigue was measured by comparing data between these 2 days. Imaging was conducted on a 3 T Siemens Trio system fitted with a 12-channel head coil. A 1,024 × 768 pixel DLP system back projected a computer display onto a front-silvered mirror, which reflected the image onto a screen. To induce LF to forearm flexors, hand contractions were performed using a Cando Rehabilitation Handgrip Exercising Device. All participants reported significant sensations of fatigue during and following the fatiguing session. This was confirmed in a more systematic experiment involving reductions in force and increases in RPE prior to the start of the experiment.

Each day, participants first performed a bicycle ergometer ride. Next, there were three consecutive fMRI scanning sessions (each 616 s) during which subjects performed repeated hand grip contractions. The three sessions tested the cortical effects of localized muscle fatigue in the forearm flexor muscles. fMRI data were collected while participants completed four tasks per experiment, each of which contained three sessions as described below: either left (L) or right (R) hand—Ƒ fatiguing hand contraction task and—Ɲ non-fatiguing hand contraction, visual stimulation (Ṿ), and rest (Ṛ) task. All subjects performed contractions with arms by their side and hands internally rotated where their palms faced the side of their upper leg. The fatiguing hand contraction task and the—Ɲ non-fatiguing hand contraction tasks were then nested as follows. In the first scanning session (before fatigue—Ḃ), fMRI data were recorded while participants contracted both of their hands at a resistance of 30% of the participant’s MVC. In the second (LF) session, we fMRI data were recorded while the participants performed fatiguing contractions at a resistance of 60% MVC. In this LF session, the RH was fatigued in experiment 1 and LH in experiment 2. The third (after fatigue—Ȧ) session was identical to the first (before fatigue—Ḃ) session with one crucial difference; forearm flexors in one hand were now fatigued despite the fact that the required force was matched. Figure 1 is a schematic of the study design.

Both hands performed the same number of contractions at an identical 30% MVC in the before fatigue (Ḃ) and after fatigue (Ȧ) sessions. This equivalence in contraction number and difference in intensity allows for a comparison of the effect of LF on hand contraction while controlling for the effect of contraction intensity. To induce LF in the RH in experiment 1 and the LH in experiment 2, they were contracted at 60% MVC. With this, one of the hands was now fatigued in the after fatigue session in each scanning experiment.

Throughout imaging, participants were given instructions on the LCD that dictated the proper task by the direction the orientation of an arrow. Left- and right-oriented arrows prompted contraction of the respective hand contractions for each Ƒ—fatiguing and Ɲ—non-fatiguing conditions. An upward-facing arrow required them to fixate on the arrow but not contract either hand—visual stimulation condition (Ṿ). A static fixation cross (Ṛ) required sole fixation on the cross. Each block was 12 s long.

Each of the three sessions included 12 blocks of all four tasks and therefore for each session there were 72 contractions per hand. These blocks were presented pseudorandomly. Participants’ contractions were visible from the MRI console and they were monitored in order to ensure that the participants were following instructions.

### fMRI Acquisition

Functional magnetic resonance imaging was collected with a T2* EPI pulse sequence using the following parameters TR = 2.2 s, TE = 30 ms, flip angle = 90°, 36 axial slices, voxels were 3 mm × 3 mm × 3 mm, with a 0.6 mm gap between slices, with 3.6 mm between slice centers. A total of 282 volumes were acquired during each session. Field maps were acquired prior to fMRI acquisition of each session, using a gradient echo sequence with TE = 5.19 and 7.65 with identical slice parameters and positioning as the subsequent fMRI sequence.

## Data Analysis

### Evaluation of Fatigue Protocol

In order to confirm the effectiveness of the handgrip contraction protocol in producing fatigue, we compared MVC force in 11 volunteers before beginning the actual experiment. There was a 13.5% (±3.4, *P* < 0.001) decline in MVC from beginning to end of handgrip protocol when contractions were performed at 60% MVC. There was no decline contractions were performed at 30% MVC. Along with this, the RPE scores using the Borg scale showed a significant change from baseline of 6 to 14.4 (±0.47) for contractions performed at 60% MVC compared to 6 to 11.1 (±0.51) for 30% MVC, respectively (*P* < 0.001).

### fMRI Analysis Tools

First level fMRI data analysis was performed on each participant’s data using FEAT (fMRI Expert Analysis Tool) Version 5.91, which is part of FSL (FMRIB’s Software Library, www.fmrib.ox.ac.uk/fsl). The pre- processing included motion correction; non-brain removal; spatial smoothing using a Gaussian kernel of FWHM 8.0 mm; grand-mean intensity normalization of the entire 4D dataset by a single multiplicative factor; and high-pass temporal filtering (Gaussian-weighted least-squares straight line fitting, with sigma = 42.5 s). EPI images were unwarped with their respective field maps using FSL’s FUGUE tool. Time-series statistical analyses were carried out with local autocorrelation correction (11). *Z* (Gaussianised T) statistic images were initially thresholded for *Z* > 2.3, with the surviving clusters subjected to a corrected cluster significance threshold of *P* = 0.05 (12) which controls for family-wise error as estimated with random field theory. Registration to the MNI 152 template was carried out using FSL’s FLIRT tool. Higher level mixed effects analysis was conducted using FLAME (FMRIB’s Local Analysis of Mixed Effects) 1 and 2 (for detailed technical reports on all of the statistics used in each of the abovementioned tools, we used in our primary fMRI analysis see http://www.fmrib.ox.ac.uk/analysis/techrep/index.html).

### First Level, Within-Session, Contrasts

Our initial analysis was for first level, within-session, contrasts: fatigued hand contractions (Ƒ), non-fatigued hand contractions (Ɲ), visual control task (Ṿ), and rest task (Ṛ). Of these the contrast [Ƒ] > [Ɲ,Ṿ] would yield the areas of the brain that were more active during Ƒ (contraction of the fatigued hand) than Ɲ (contraction of the non-fatigued hand) and Ṿ (visual fixation).

To investigate our hypothesis about drift influencing related literature, we also conducted 12 other within-session contrasts. The 13 total within-session contrasts we performed are as follows: [Ƒ], [Ɲ], [Ṿ], [Ɲ] > [Ṿ], [Ƒ] > [Ṿ], [Ṿ] > [Ɲ], [Ṿ] > [Ƒ], [Ƒ,Ɲ] > [Ṿ], [Ṿ] > [Ƒ,Ɲ], [Ɲ,Ṿ] > [Ƒ], [Ƒ,Ṿ] > [Ɲ], [Ɲ] > [Ƒ,Ṿ]. All first level, within-session, contrasts were conducted with FEAT.

### Second Level, Between-Session Contrasts

Each day, every participant completed three scanning sessions. Here again, we refer to these three sessions as before fatigue—Ḃ, LF and after fatigue—Ȧ. In order to measure the cortical activity that might represent the effect of localized hand fatigue on the brain, we used FLAME to conduct paired two-group difference contrasting all of the before fatigue—Ḃ sessions vs. all of the after fatigue—Ȧ sessions (two-sample paired *T*-tests, http://www.fmrib.ox.ac.uk/fsl/feat5/detail.html#PairedTwoGroupDifference).

We surmised that the physical work load was matched between Ḃ and Ȧ. To detect any differences of cortical activity between the two nevertheless we performed higher level mixed effects contrast using two-sample paired *T*-tests between the first level fatigue contrasts ([Ƒ] > [Ɲ,Ṿ]) between Ḃ and Ȧ. This is represented as follows; ([ḂƑ] > [ḂƝ, ḂṾ]) > ([ȦƑ] > [ȦƝ, ȦṾ]) and ([ȦƑ] > [Ȧ Ɲ, ȦṾ]) > ([ḂƑ] > [ḂƝ, ḂṾ]). These higher level contrasts are explorations of cortical activity for identical hand contraction forces, compared before LF was induced compared to after fatigue locally, but before recovery.

To detect the cortical representation of generalized full body fatigue, the comparisons would be between easy ride—Ẹ vs. hard ride—Ḥ. To arrive at this, the formula is given by ([ẸƑ] > [ẸƝ, ẸṾ]) > ([ḤƑ] > [ḤƝ, ḤṾ]), and ([ḤƑ] > [ḤƝ, ḤṾ]) > ([ẸƑ] > [ẸƝ, ẸṾ]).

### Drift Detection

**Figure 2:**
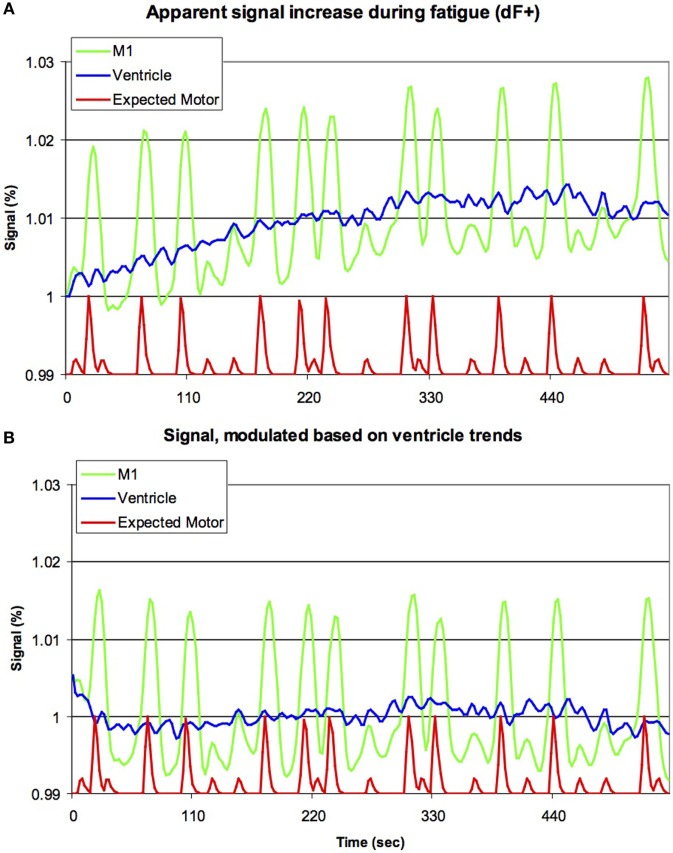
Previous studies have suggested that brain activation increases during fatigue (dF+). Our raw data for the initial 255 volumes [upper panel, **(A)**] appears to support this notion, with the signal from left primary motor cortex (M1, green line) increasing with time (horizontal axis), though clearly this signal is also modulated by the movement of the hands (red line, smoothed by the sluggish nature of the blood-flow’s response, with large and small bumps indicating right and left hand movements, respectively, though note this line is shown offset and with reduced amplitude). However, careful inspection casts doubt on this conclusion, as a very similar signal increase is seen for the ventricles (blue line). As the ventricles are non-vascular, this suggests that this progressive signal increase is due to artifacts, rather than changes in metabolism. The lower panel **(B)** is identical to the top panel, except that the data have been modulated based on the signal of the ventricles (for each individual, the signal change in the ventricles was modeled using a power function, and subsequently the image for the entire brain was rescaled to remove this effect). Note that the activity in M1 is still influenced by hand movements, but there is no evidence for a continuous signal increase. This suggests that signal artifacts are sufficient to explain dF+.

In order to test that the signal changes that would be detected form statistical contrasts dFEX occurred uncontrolled artifacts as may be the case in the previously reported studies and not from true brain activity, we analyzed LF sessions in detail. First, we replicated the methods used in prior literature, allowing us to replicate the (dFEX) finding (Figure 2A). We also extended previous work by modeling signal changes in the cerebral spinal fluid (CSF) based on the premise that CSF contains no brain tissue nor a vascular supply and therefore any signal changes must reflect signal drift. We then investigated whether the changes in the MR signal detected in the CSF space were sufficient to describe the progressive change seen in the cortical areas as the scanning progressed over time. We applied a power function to fit the signal we observed in a region of interest (ROI) in the middle of each individual’s ventricle, then scaled the data observed throughout the brain based on this function. In effect, this power function acted as a high-pass temporal filter tuned to the changes observed in the CSF space within the ventricles that had no physiological surrogates.

### Habituation Detection

One major concern emanating from the prior studies in the literature is the possibility of effects of habituation. To rule out this possibility, we performed a higher level mixed effects two-sample paired *T*-test analysis. The resulting higher level between-sessions contrasts take the form of ([Ḃ Ɲ] > [ḂṾ]) > ([ȦƝ] > [ȦṾ]) and ([Ḃ-Ƒ] > [ḂṾ]) > ([ȦƑ] > [ȦṾ]).

### Regions of Interest

Functional regions of interest were created in order to investigate the percentage of BOLD signal that changed pre- and postfatigue. Regions were created using the statistical maps generated. The values consisted of averages across regions, defined by the statistical contrasts from experiment 1 using featquery, which is part of FSL (FMRIB’s Software Library, www.fmrib.ox.ac.uk/fsl).

Experiment 2 was conducted several weeks following experiment 1 to examine possible laterality effects that may have occurred in the initial experiment. The only change in the protocol was fatiguing of the LH rather than RH.

## Results

### Drift Detection

Analysis of LF sessions using analyses identical to previous literature replicated a progressive signal increase dFEX conditions in both cortical tissue as well as within CSF space (Figure 2A). We modeled the signal change in CSF with a power function and used this value to scale the image intensity throughout the brain. This modeling correction for signal drift fully eliminated signal changes in the cortical regions (Figure 2B).

### Cortical Effects of Habituation during Exercise

**Figure 3:**
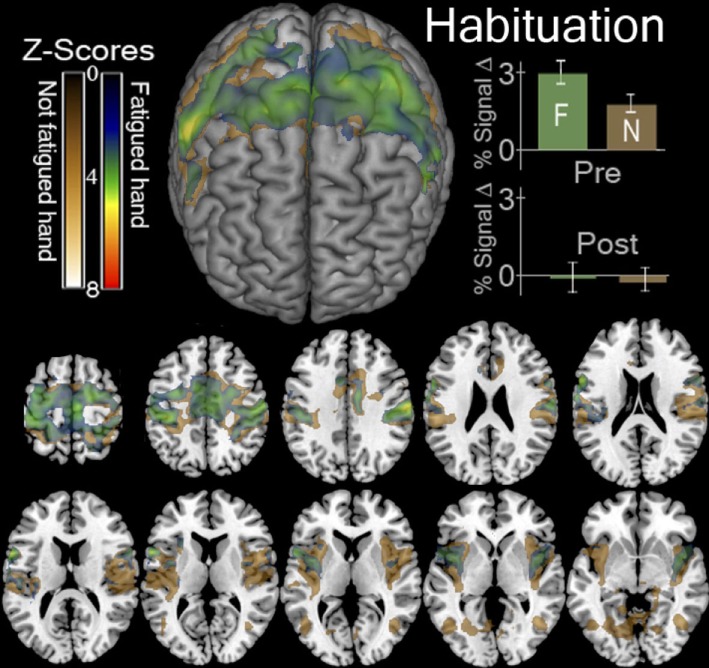
Functional magnetic resonance imaging (fMRI) contrasts showing regions that were more active in the first session than the last session when contracting a hand > looking at the instruction arrow. This contrast for the fatigued hand or Ƒ, ([ḂƑ] > [ḂṾ]) > ([ȦƑ] > [ȦṾ]) is shown in green. This contrast for the hand that was not fatigued or Ɲ, ([ḂƝ] > [ḂṾ]) > ([ȦƝ] > [ȦṾ]) is shown in gold. In the top left we display a surface rendering of the significant regions looking at the front of the brain from above. Underneath and to the right of this image are axial slices displaying these contrasts. The slices correspond to *Z* values of −5, −10, 0, 5, 10, 15, 20, 25, 30, 45, 60 in MNI stereotaxic space. In the top right we display the percent of signal change (mean ± SEM) observed in the regions indicated by these contrasts in the first, before-local fatigue, session and in the third, after local fatigue. We illustrate the enormous decrease in signal thought the motor cortex for both hands regardless of fatigue.

The contrasts resulting from [Ƒ] > [Ṿ] and [Ɲ] > [Ṿ] was larger in the before fatigue—Ḃ sessions than the—Ȧ sessions. This occurred throughout the motor system. As we hypothesized, the contrasts ([ḂƝ] > [ḂṾ]) > ([ȦƝ] > [ȦṾ]) and ([ḂƑ] > [ḂṾ]) > ([ȦƑ] > [ȦṾ]). Both revealed declining activation throughout the bilateral cortical motor system as the locally fatiguing exercises during scanning progressed (Figure 3). ROI Analysis of the visual cortex revealed a similar decline (Figure 3).

### Cortical Activity Representing Full Body Generalized Fatigue

**Figure 4:**
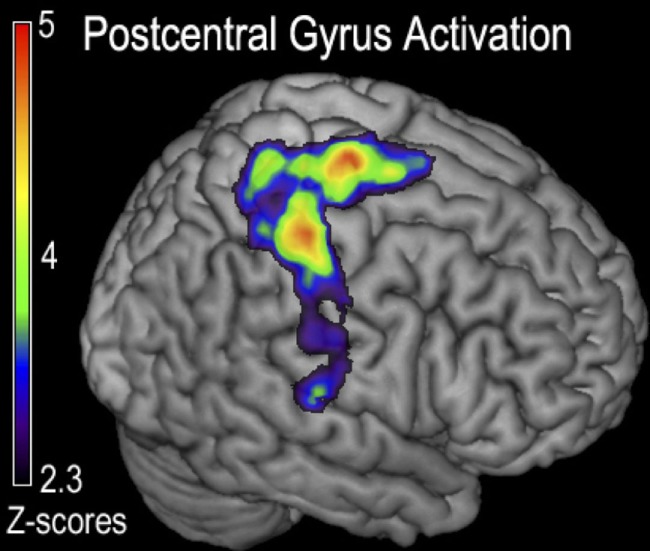
Functional magnetic resonance imaging (fMRI) contrast of postcentral gyrus activation observed for the generalized fatigue ([ḤƑ] > [ḤƝ,ḤṾ]) > ([ẸƑ] > [ẸƝ,ẸṾ]) contrast in experiment 1. This contrast corresponds to greater activity after generalized fatigue. Regions indicated correspond to the majority of the postcentral gyrus, with a peak just posterior to the cortex responsible for the control of the fatigued hand, as well as a peak in the M1 corresponding to the primary motor control of the lower extremity that was fatigued during cycling.

The hard ride > easy ride seeks to answer the question of generalized full body fatigue by comparing fatigue contrast-related activity that was greater following a hard cycle ride than an easy ride. This contrast ([ḤƑ] > [ḤƝ, ḤṾ]) > ([ẸƑ] > [ẸƝ, ẸṾ]) revealed a large cluster of activity mainly weighted in the dorsal third of the right precentral gyrus, including right Brodmann Areas 48 (aPMv), BA6, BA43 and as well as the insular cortex and parts of the right postcentral gyrus (*P* < 0.05, corrected) (Figure 4; Table 1).

**Table 1:** The cluster of activation in which greater activation was observed for the generalized fatigue hard more than easy ride ([ḤƑ] > [ḤƝ, ḤṾ]) > ([ẸƑ] > [ẸƝ, ẸṾ]) contrast in experiment 1.

Cluster volume (cm^3^)		*Z* score	MNI coordinates (*x, y, z*)	Location	Broadmann areas
1	38.44	5.14	62, −16, 32	R post central gyrus	43
		4.96	−40, −28, 51	L post central gyrus	3
		4.68	−58, 8, 18	L frontal operculum	6
		4.51	21, 9, 54	R superior frontal gyrus	6
		4.11	0, −19, 52	SMA	6
		3.65	44, 2, 2	R insula	48

*This contrast corresponds to greater activity after generalized fatigue. Threshold: Z > 2.3, corrected cluster threshold P < 0.05. Given that the tasks are being performed with R hand, right side is ipsilateral. This cluster is rendered in Figure 4*.

### Cortical Effects of Localized Hand Fatigue

**Figure 5:**
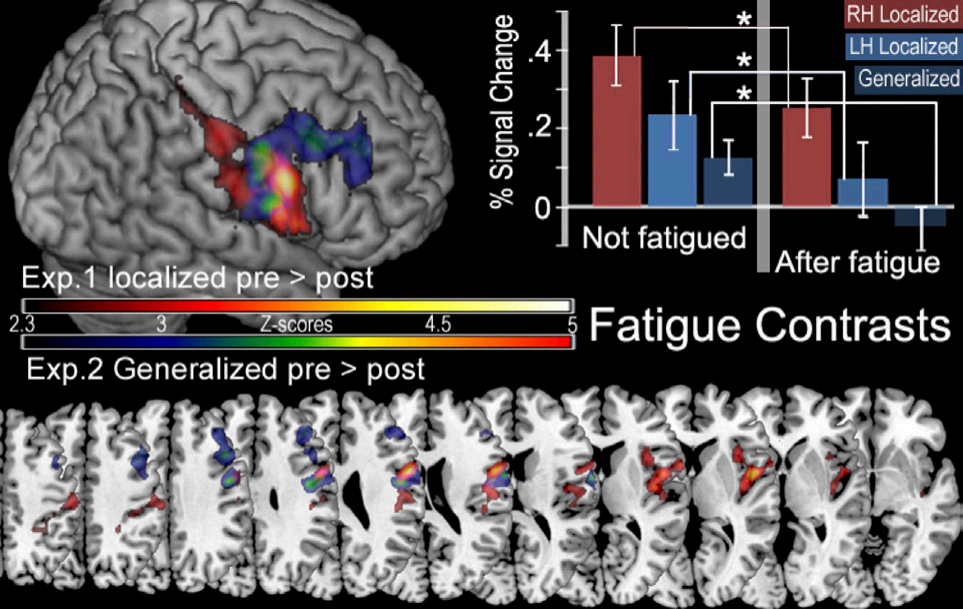
In the top left corner is a surface rendering of regions more active before localized fatigue ([ḂƑ] > [ḂƝ, ḂṾ]) > ([ȦƑ] > [ȦƝ, ȦṾ]) in experiment 1 (red) and regions more active when participants were not experiencing generalized fatigue ([ẸƑ] > [ẸƝ, ẸṾ]) > ([ḤƑ] > [ḤƝ, ḤṾ]) in experiment 2 (blue). Below this are the color gradients used to illustrate the significance of the contrasts. The bottom of the figure displays overlapping slices illustrating these contrasts. The location of these slices corresponds with *Z* values of −5, −10, 0, 5, 10, 15, 20, 25, 30, 45, 50 in MNI stereotaxic space. There are no significant clusters of activity in the left hemisphere. In the top right corner, we display the percent of signal change (mean ± SEM) observed in the aMPv in the presence and absence of fatigue. Here we show significantly less activity after fatigue in the aPMv of the right hemisphere while contracting the right hand (RH), in the left aPMv after fatiguing the left hand (LH), as well as less activity in the right aPMv following generalized fatigue.

Cortical activity that results from inducing LF was detected with the contrast ([ḂƑ] > [ḂƝ, ḂṾ]) > ([ȦƑ] > [ȦƝ, ȦṾ]) which revealed, significant decline in the BOLD signal that was located in the right postcentral sulcus at the intersection of right Brodmann Areas 6, 43, and 48 [anterior ventral premotor cortex (aPMv)] (see Figure 5 and Table 2). No other between-session contrast resulted in significant activation.

**Table 2:** The significant cluster of activation revealing brain areas that were significantly more activated for the before-LF vs. the after local-fatigue contrast in experiment 1.

Cluster volume (cm^3^)	*Z* score	MNI coordinates (*x, y, z*)	Location	Broadmann areas
17.44	4.5	62, 12, 10	R rolandic operculum	6
	4.14	56, 6, 24	R precentral gyrus	6
	4.02	49, 4, 6	R rolandic operculum	48
	3.41	36, 16, 8	R insula	48
	3.24	41, 9, 8	R insula	48
	3.09	50, −20, 44	R postcentral gyrus	4

*This is brain activation found to be more correlated with contracting the fatigued hand more than the non-fatigued hand and the visual cortex, before more than after fatigue ([ḂƑ] > [ḂƝ, ḂṾ]) > ([ȦƑ] > [ȦƝ, ȦṾ]) Threshold: *Z* > 2.3, corrected cluster threshold *P* < 0.05. Given that the tasks are being performed with R hand, Right side is ipsilateral. This cluster is rendered as the red cluster in Figure 5*.

### Laterality Effects of LF

Region of interest analysis created by the contrast for after fatigue —Ȧ sessions compared to before fatigue—Ḃ sessions given by ([ḂƑ] > [ḂƝ, ḂṾ]) > ([ȦƑ] > [ȦƝ, ȦṾ]) found a significant reduction in the BOLD signal intensity in the in the aPMv of the left (ipsilateral) hemisphere (df = 4, *P* = 0.043) when first experiment was compared to the second. There were no such activations in the right (contralateral) hemisphere (df = 4, *P* = 0.94). On the other hand, the ROI analysis also revealed a significant activation in the aPMv of the right hemisphere when cortical activity subsequent to easy ride was compared to the hard ride ([ẸƑ] > [ẸƝ, ẸṾ]) > ([ḤƑ] > [ḤƝ, ḤṾ]) (Figure 5; Table 3).

**Table 3:** The significant cluster of activation revealing brain areas that were significantly more activated when participants were not experiencing generalized fatigue ([ẸƑ] > [ẸƝ, ẸṾ]) > ([ḤƑ] > [ḤƝ, ḤṾ]) more than when they were, in experiment 2 threshold: *Z* > 2.3, corrected cluster threshold *P* < 0.05. This cluster is rendered as blue cluster in Figure 5. R side is ipsilateral.

Cluster volume (cm^3^)	*Z* score	MNI coordinates (*x, y, z*)	Location	Broadmann areas
17.37	3.75	62, 2, 18	R postcentral gyrus	48
	3.58	42, 6, 30	R postcentral gyrus	44
	3.42	40, 40, 30	R middle frontal gyrus	46
	3.29	46, 20, 35	R frontal operculum	44

## Discussion

### Cortical Substrate of Supra-Spinal Fatigue

In this study, our main finding is a statistically significant fatigue-related decline in the metabolic demand of a large cluster whose main volume was contained within the ipsilateral aPMv and the adjacent cortical regions. Both localized and generalized effects of fatigue were lateralized to the ipsilateral (side of the hand being fatigued) hemisphere. aPMv has been identified as a possible fatigue center in previous brain imaging studies of localized fatigue (18, 19). However, while those previous imaging research focused only on neural representation of localized fatigue, we now report cortical activity that is specifically associated with localized as well as generalized fatigue.

The significance of our findings emanates from the previously well known neuroanatomical and neurophysiological traits of the regions we have identified. For example, the ventral premotor cortex (PMv), just posterior to the aPMv is involved in motor execution in monkeys is responsive to differing intensities of force vectors (20). This region is considered a physiological and a phylogenetic motor planning homolog in the human brain (21). With this, it is well known that the aPMv output is related to motor activity. It has ubiquitous connections to the ventral and central caudate nucleus, ventral striatum, and insula (22), all of which are involved with mood and initiation of motor control. Many segments of this circuitry have been implicated to effect and be effected by muscle fatigue (7). In addition, other areas of the prefrontal cortex, such as the ventrolateral prefrontal cortex (VLPC), just anterior to the aPMv, have been implicated in cognitive inhibition of behavior (23). aPMv has been recently shown to be modulated during psychological perception of fatigue (24). Moreover, transcallosal inhibition of contralateral motor cortex by corresponding areas of ipsilateral motor cortex during motor behavior is a well-known phenomenon, and alteration in this inhibitory process has been shown to contribute to supraspinal fatigue (25–27). Another study that used TMS over the PMv causes a disruption in the control of the force of handgrip contractions (28), and corticospinal motor output from M1 to contralateral hand muscles can be facilitated or inhibited by TMS over the ipsilaeral PMv (29). Given that neural output from M1 is thought to be reduced during fatigue (7), and given the M1 and PMV connectivity, it seems reasonable to surmise that the ipsilateral aPMv is responsible for inhibiting the output of M1 in order to reduce the force of muscle contraction during fatigue. aPMv may induce supraspinal fatigue by altering intracortical inhibition from the ipsilateral motor cortex, with which it is densely connected (22). And a recent study using optical imaging suggested that the subjective rating of psychological fatigue correlates with reduced activation in the VLPC (24). In addition, an fMRI study recently found that the delay in fatigue during cycling that results from carbohydrate sensing in the mouth was associated with activation of the dorsolateral premotor cortex (30). Taken together these studies using multiple experimental techniques and paradigms all strongly point to the aPMv as a common neural substrate for fatigue, regardless of its origin.

Many fundamental question about fatigue remains unanswered to date. The present study extends the previous knowledge on neural substrates of localized fatigue to that of generalized fatigue and provides the first of compelling support for the existence of a prolonged effect of exercise on the cortical motor system, largely localized to the ipsilateral aPMv.

Another finding of our experiment was that an exhaustive fatiguing induced a greater somatosensory activation of the dominant hemisphere, for example, when—Ḥ, the hard bicycle ride was compared to an—Ẹ, the easy bicycle ride. Given that the somatosensory cortex receives input from type III and IV muscle afferents and this finding may reflect the differential processing of non-fatigued muscles. It is plausible that increased metabolic demand in the postcentral gyrus and the superior regions of the precentral gyrus that control the muscles are most active during cycling, leading to a lasting effect on the precentral gyrus’ role in fatigue.

### Replication and Explication of Previously Reported Findings

By applying temporal filtering and using hierarchical contrasts, our study paradigm and analyses obtained unbiased evidence of fatigue-related brain activity. Here, we have been able to show that previous findings reflect drift rather than true brain activity by conducting an analysis without these filters. Specifically, when modeled without temporal filtering, the within-session analysis of our second scanning session (LF) we noted and replicated the previously reported effect of a progressive signal increase dFEX. However, this effect was not limited to the cortical substrate but was also notable in non-metabolic regions, such as the CSF space (upper panel of Figure 2). Signal changes in this region must reflect signal drift, rather than true changes in metabolism that are detected by BOLD signals. In theory, it is remotely possible that the cortical signals we observed could reflect both signal drift and true cortical activity of dFEX. To tease apart these factors, we modeled the signal change in the CSF and adjusted the image intensity throughout the brain to compensate for this change. Following this adjustment, there was no longer a within-session increase in the motor cortex (lower panel of Figure 2) thereby eliminating that unlikely scenario.

Previous exercise imaging studies (5, 11, 31, 32) had reported data suggestive of decreased cerebral metabolism aFEx. We replicated this effect and observed a similar decline in the visual cortex (Figure 3). The lack of specificity of signal drop seen in visual cortex suggests that this is not a biomarker for fatigue.

In summary, our data suggest that ipsilateral aPMv is a likely candidate for a cortical nexus of central fatigue. It may function this way to mediate a reduction in the neural output from the contralateral M1 by modulating intrahemispheric transcallosal inhibition from the ipsilateral M1. We observed higher BOLD activity in the aPMv before fatigue occurs than after fatigue was induced, independent of whether it was generalized or localized. We also report increased activity in the postcentral gyrus and M1 following exhaustive generalized fatigue that we attribute to heightened processing of sensory information from type III and IV muscle afferents. Future imaging and neurophysiological experiments with repetitive. TMS might allow a path to test these findings further and perhaps pave a way to the development of non-pharmacologic, non-invasive therapeutic measures to help with sportsmen, soldiers, others who are engaged in physically demanding work in their occupations and perhaps even patients who happen to face functional limitations due to severe fatigue.

There are some limitations to the study. One is that this was conducted on normal healthy young volunteers, whose fatigue thresholds are probably higher compared to patients. As the volunteers were young and healthy, sleep issues were unlikely to have been a confounding issue for fatigue during task performance. However, we did not explicitly ask about nocturnal sleep problems that may cause or worsen fatigue (e.g., sleep apnea, insomnia, poor quality sleep, etc.). Likewise, we did not measure individual differences in mental fatigue which is associated with central fatigue (33). Investigating these factors would be an interesting avenue for future research. While the design of the experiment used a logical approach to eliminate training and habituation effects, it is impossible to tell whether this was fully achieved. Another issue might pertain to our statistical thresholding. We are aware of the recent controversial paper (34). Although their simulations demonstrated that false positives can be elevated when using cluster-level inference, they stress that their effects are spatially dependent (e.g., more likely in some regions) and are exacerbated when assumptions are not met. In addition, their results also show that the Bayesian mixed effects approach utilized in FSL’s FLAME1 does not result in elevated false positives, and indeed note the “highly conservative results from FSL FLAME1.” We emphasize that this is the approach we used in our article. Others have since used the same methods (35), thus validating our approach. Additionally, in order to increase sampling rate, we used a small number of slices, and therefore we were unable to detect signals in the cerebellum. This concern could be addressed in future studies, potentially leveraging recent advances in multiband imaging that allow more slices to be acquired in a given sampling period. We believe that further studies with other methods including simulations and different models of fatigue may be necessary to parse this issue.

While our primary goal was to detect central (brain) changes in response to fatigue, these central changes may in fact interact with peripheral fatigue—one could think of stronger signals to drive weakened peripheral muscles as well as attenuated signals to prevent injury. Regardless, our findings do illustrate that clear changes are detected after fatigue.

We acknowledge that our main findings are based on easy motor tasks conducted after localized or generalized fatigue. A consequence of this paradigm is that it is hard to determine whether the effects reflect fatigue *per se* or rather recovery following fatigue. However, while our inferences are limited, our design does match the physical exertion and captures the enduring aspects of the fatigue and recovery seen in the real world.

## Ethics Statement

This study was carried out in accordance with the recommendations of University of South Carolina Institutional Review Board (https://www.healthsciencessc.org/initiative/electronic-institutional-review-board-eirb/236) with written informed consent from all subjects. All subjects gave written informed consent in accordance with the Declaration of Helsinki. The protocol was approved by the University of South Carolina Institutional Review Board.

## Author Contributions

All authors: Substantial contributions to or the acquisition, analysis, interpretation of data for the work; drafting the work or revising it critically for important intellectual content; final approval of the version to be published; and agreement to be accountable for all aspects of the work in ensuring that questions related to the accuracy or integrity of any part of the work are appropriately investigated and resolved.

## Conflict of Interest Statement

The authors declare that the research was conducted in the absence of any commercial or financial relationships that could be construed as a potential conflict of interest. The reviewer PP and handling editor declared their shared affiliation.
